# Bruton’s Tyrosine Kinase Mediates the Synergistic Signalling between TLR9 and the B Cell Receptor by Regulating Calcium and Calmodulin

**DOI:** 10.1371/journal.pone.0074103

**Published:** 2013-08-14

**Authors:** Elaine F. Kenny, Susan R. Quinn, Sarah L. Doyle, Paul M. Vink, Hans van Eenennaam, Luke A. J. O’Neill

**Affiliations:** 1 Immunology Research Centre, School of Biochemistry and Immunology, Trinity Biomedical Sciences Institute, Trinity College Dublin, Dublin, Ireland; 2 School of Biochemistry and Immunology, Trinity Biomedical Sciences Institute, Trinity College Dublin, Dublin, Ireland; 3 Janssen Infectious Diseases BVBA, Beerse, Belgium; 4 BioNovion B.V., Oss, The Netherlands; University of Miami, United States of America

## Abstract

B cells signal through both the B cell receptor (BCR) which binds antigens and Toll-like receptors (TLRs) including TLR9 which recognises CpG DNA. Activation of TLR9 synergises with BCR signalling when the BCR and TLR9 co-localise within an auto-phagosome-like compartment. Here we report that Bruton’s tyrosine kinase (BTK) is required for synergistic IL6 production and up-regulation of surface expression of MHC-class-II, CD69 and CD86 in primary murine and human B cells. We show that BTK is essential for co-localisation of the BCR and TLR9 within a potential auto-phagosome-like compartment in the Namalwa human B cell line. Downstream of BTK we find that calcium acting via calmodulin is required for this process. These data provide new insights into the role of BTK, an important target for autoimmune diseases, in B cell activation.

## Introduction

The B cell receptor (BCR) is a multi-protein complex containing an immobilised immunoglobulin (mIg) found on the plasma membrane of B cells that is responsible for the recognition of antigens by B cells [1,2]. Many B cells patrol the blood and lymphatic system searching for potential pathogens or pathogenic molecules. Each B cell expresses a unique BCR generated to interact with one specific antigen and as such they ensure a rapid response when an infection is encountered [3,4]. Once the BCR has been activated a signal transduction pathway is initiated within the B cells and a highly specific antibody response occurs that targets the pathogen for phagocytosis and allows activation of the complement cascade [1]. Internalisation of the BCR ensues subsequent to antigen binding, transporting the BCR to a MHC-class-II containing compartment within the B cells [5,6]. This allows antigen presentation to T cells by the B cell and ensures further assistance in pathogen clearance by T cell-dependent responses.

Whilst the BCR is central to B cell function it is becoming more evident that many other co-stimulatory receptors found on the plasma membrane and within endosomes of the B cells aid in the regulation of B cell signalling [7]. Of these co-stimulatory molecules the Toll-like receptors (TLR), a family of proteins central to innate immune signalling, are of great interest due to the link between TLR and BCR signalling in autoimmunity [8]. Recent studies investigating BCR and TLR signalling has revealed that TLR9, which recognises double stranded poly-unmethylated CpG DNA motifs in bacteria and viruses [9], or in autoimmunity host DNA, synergises with the BCR leading to enhanced signal transduction. One such study demonstrated that synergy occurred due to the translocation of TLR9 and the BCR to an auto-phagosome-like compartment upon BCR activation. This translocation was shown to be microtubule-dependent and was hypothesised to allow optimal antigen presentation by activated B cells since markers of MHC-class-II molecules such as the invariant chain were also localised within this auto-phagosome-like compartment [10].

Bruton’s tyrosine kinase (BTK) is a member of the Tec family of protein-tyrosine kinases (PTKs) [11] that is known to be required for both TLR9 and BCR signal transduction [12,13]. BTK was first identified as the gene responsible for X-linked agammaglobulinaemia (XLA) in humans which is characterised by severe defects in early B cell development with a near complete absence of peripheral B cells and immunoglobulin’s of all classes [14]. A similar condition is found in mice with a naturally occurring mutation at arginine 28 (R28C) in the pleckstrin homology domain of BTK which results in the development of X-linked immune deficiency (Xid) [15].

In response to crosslinking of the BCR BTK becomes recruited to the plasma membrane via its pleckstrin homology domain and becomes phosphorylated and activated. It then phosphorylates its target phospholipase C-gamma 2 (PLC-γ2) which in turn cleaves phosphatidylinositol 4,5-bisphosphate (PIP-2) into diacylglycerol (DAG) and inositol trisphosphate (IP-3) [1]. The generation of IP-3 leads to the release of calcium (Ca^2+^) from the endoplasmic reticulum through its interaction with the IP-3 receptor [16]. This increase in cytosolic Ca^2+^ also results in a further influx of Ca^2+^ from the extracellular matrix via store-operated Ca^2+^ entry (SOCE) [17,18]. It is well established that this Ca^2+^ flux plays a vital role in BCR signal transduction by inducing transcription factors such as NFAT and NFκB that regulate immune functions, cell differentiation and proliferation [19]. The increase in cytosolic Ca^2+^ along with the activation of many other signalling molecules in response to BCR stimulation ensure the full activation of the B cells and the subsequent maturation and differentiation of the B cells culminating in the antibody response [4].

In TLR signalling BTK has been shown to interact with several TLRs and TIR domain containing adapter proteins [20,21]. It is also involved in the phosphorylation of the p65 component of NFκB in response to TLR4 stimulation [22]. A direct interaction between TLR9 and BTK has been described in THP-1 cells and this interaction is necessary for TLR9 signalling. Peripheral blood mononuclear cells (PBMC) from XLA patients are impaired for cytokine production in response to CpG [13]. BTK has also been demonstrated to be required for optimal IL12 and IL10 production and phosphorylation of p65 in response to CpG in B cells [12].

We utilised a highly specific small molecule inhibitor of BTK, PCI-32765 [23], to investigate the role of BTK in TLR9 and BCR synergy. We have found that BTK is essential for TLR9 and BCR synergy from an auto-phagosome-like compartment, acting to increase calcium which in turn acts via calmodulin. We have therefore elucidated a critical role for BTK, an important target for autoimmunity [24], in B cell co-stimulation.

## Materials and Methods

### Ethics statement

Wild-type (WT) C57BL/6 mice were obtained from Harlan, UK and maintained in a specific-pathogen free environment. All animal experiments were performed in compliance with Irish Department of Health and Children regulations and approved by the Trinity College, Dublin’s BioResources ethical review board.

### Isolation of primary B lymphocytes

Human B cells were purified from buffy coat PBMCs supplied by the Irish Blood transfusion service using a positive selection protocol with anti-CD19 mAb-coupled magnetic beads (Miltenyi Biotec). Murine splenic B cells were isolated from spleens of wild type C57BL/6 mice using a negative selection protocol with anti-CD43, CD4 and Ter-119 mAb-coupled magnetic beads (Miltenyi Biotec). Primary B cells were cultured in RPMI-glutamax (Life Technologies) supplemented with 10% Fetal calf serum (FCS) (Sigma) and 1% penicillin-streptomycin (Sigma).

### Reagents and cell lines

F(ab’)_2_ goat anti-mouse and F(ab’)_2_ rabbit anti-human antibodies specific for mouse and human IgM (anti-IgM) and the Alexa fluor-647 anti-IgM were purchased from Jackson Immnuo Research Laboratories. CpG-ODN 1826, CpG ODN 2006, CpG ODN 2006-FITC and LPS were from Invivogen. Ionomycin, thapsigargin and the IP-3 receptor inhibitor Xestospongin C were from Sigma. The PLC-γ2 inhibitor U-73122 was from Calbiochem. The BTK inhibitor PCI-32765 was synthesised by Organon International (Netherlands) and is available from Selleck Chemicals (Houston, Texas, USA). The calmodulin inhibitor W7 was purchased from Santa Cruz. The FITC-MHC-class-II, FITC-IgG isotype control, CD69 and CD86 antibodies were from BD Pharmingen. Fluo-3AM and BAPTA-AM were purchased from Invitrogen. The phospho-PLC-γ2 antibody was from Cell Signalling Technologies. The Namalwa human B cell line was purchased from ATCC (VA, USA) and maintained in RPMI-glutamax supplemented with 10% FCS and 1% penicillin-streptomycin.

### IL6 ELISA

Primary human and murine B cells were cultured at 1x10^6^ cells/ml in 96 well plates, pre-treated with 1-50 nM BTK inhibitor, 250 nM -1 µM PLC-γ2 inhibitor, 500 nM -2 µM IP-3 receptor inhibitor, 1 µM BAPTA-AM or 3 µM calmodulin inhibitor for 1 hr and stimulated with 1-5 µg/ml CpG, 10 µg/ml anti-IgM, 1 ng/ml-10 µg/ml LPS, 500 nM thapsigargin or a combination of the ligands indicated for 48 hr at 37^o^C. The concentrations of inhibitors and ligands used were chosen based on examination of the literature. Supernatants were collected and the concentration of IL6 was determined by ELISA (R&D Systems). Absorbance at 450 nm was measured on a spectrophotometric ELISA plate reader.

### MHC-II surface expression analysis

Primary human B cells were set-up at 1x10^6^ cells/ml, pre-treated with 20 nM BTK inhibitor for 1 hr, stimulated for 48 hr with 5 µg/ml CpG, 10 µg/ml anti-IgM, both or 50 U/ml IL4, centrifuged and resuspended in FACS buffer. The cells were incubated in Fc block (Miltenyi Biotec) at 4^o^C for 10 min and then stained with FITC-MHC-class-II or FITC-IgG isotype control antibodies for 20 min at 4^o^C. The cells were then washed twice in FACS buffer and analysed by flow cytometry on a FACS Calibur using FlowJo software with live cells gated by PI staining.

### B cell activation marker analysis

PBMCs were isolated from human blood, incubated with the concentrations of the BTK inhibitor indicated for 30 min and stimulated for 24hr with 10 µg/ml anti-IgM or 10 µg/ml anti-IgM plus 1 µg/ml CpG. The cells were then stained for 30 min with an anti-CD19 antibody to gate for B cells and either an anti-CD69-PE-Cy7 or CD86-PE antibodies. Cells were washed and analysed by flow cytometry on a FACS Cantoll.

### Calcium flux assay

1x10^6^ cells Namalwa B cells/point were cultured in Iscove’s Modified Dulbecco’s Medium (IMDM) and incubated with 20 nM BTK inhibitor or 1 µM BAPTA for 20 min. The cells were then incubated with 4 µM Fluo-3AM for 30 min. To quench extracellular Fluo-3AM the cells were then incubated in 10% FCS/IMDM for 10 min at room temperature. The cells were then washed twice in 5% FCS/IMDM and resuspended in Hanks’ balanced salt solution (without MgCl_2_ or CaCl_2_) supplemented with 5% FCS, 1.5 mM CaCl_2_ and 1 mM HEPES. The cells were analysed for 40 sec, stimulated with 5 µg/ml CpG, 10 µg/ml anti-IgM, both or 1 µM ionomycin and further analysed by flow cytometry on a FACS Calibur for a total of 200 sec.

### Confocal Microscopy

1x10^6^ Namalwa B cells/ dish were cultured in poly-D-lysine coated glass bottom dishes, incubated with 20 nM BTK inhibitor, 1 µM BAPTA or 3 µM W7 for 1 hr and stimulated for 16 hr with 2 µM FITC-labelled CpG, 10 µg/ml Alexa fluor-647-labelled F(ab’)_2_ rabbit anti-human IgM or both. The live cells were then imaged using an Olympus FV1000 confocal microscope. Fixed cell imaging was carried out by fixing the stimulated cells in 4% paraformaldehyde (Sigma) at room temperature for 10 min and permeabilising the cells in 0.2% triton X-100 (Sigma) for 15 min at room temperature. The cells were then blocked in 20% FCS/PBS for 1 hr, stained with an anti-EEA1 antibody (Abcam) for 2 hr at room temperature and incubated in an Alexa-flour-546-labelled F(ab’)_2_-goat anti rabbit secondary antibody (Invitrogen) for 45 min at room temperature. The cells were then examined on an Olympus FV1000 confocal microscope. Images were analysed using the Olympus FV1000 Confocal Microscope Viewer software.

### Western Blot Analysis

Primary Human or Namalwa B cells were set-up at 2x10^6^ cells/point, pre-treated with 20 nM BTK inhibitor for 1 hr and stimulated with 5 µg/ml CpG, 10 µg/ml anti-IgM or CpG and anti-IgM at 37^o^C for 30 min. The cells were lysed in high stringency lysis buffer (50 mM HEPES, pH 7.5, 100 mM NaCl, 1 mM EDTA, 10% glycerol, 1% Nonidet P-40 containing 10 µg/ml phenylmethylsulfonyl fluoride, 30 µg/ml aprotinin, 1 µg/ml sodium orthovanadate and 1 µg/ml leupeptin). The protein concentration of each was determined using Coomassie Bradford reagent (Pierce) according to manufacturer’s instructions. Normalised samples were then analysed by SDS-PAGE and immuno-blotted for the phosphorylation of PLC-γ2 and β-actin as per the manufacturer’s instructions. Densitometric analysis of relative intensities (R.I) was determined using ImageJ Version 1.46r.

### Statistical analysis

ELISA statistics: The concentration of IL6 produced by the B cells in response to CpG and anti-IgM was set to maximum response at 100% and all other stimulations were normalised to this value. This analysis was carried out on three independent experiments, each carried out in triplicate. For comparison between two groups a two tailed Student’s *t* test was used.

MHC class-II FACs statistics: Analysis was carried out on the mean fluorescence intensities (MFI) from three independent experiments. For comparison between groups a two tailed Student’s *t* test was used.

Confocal microscopy statistics: The number of FITC-CpG and Alexa 647 anti-IgM containing specks in 50 cells was compared to the number of specks found in 50 cells pre-treated with the BTK inhibitor, BAPTA or W7. A 2x2 contingency table chi squared test was then carried out and a *p* value of <0.05 was considered significant.

## Results

### Stimulation of TLR9 and the BCR results in synergistic IL6 production and BTK is required for this in primary murine B cells

**Figure 1:**
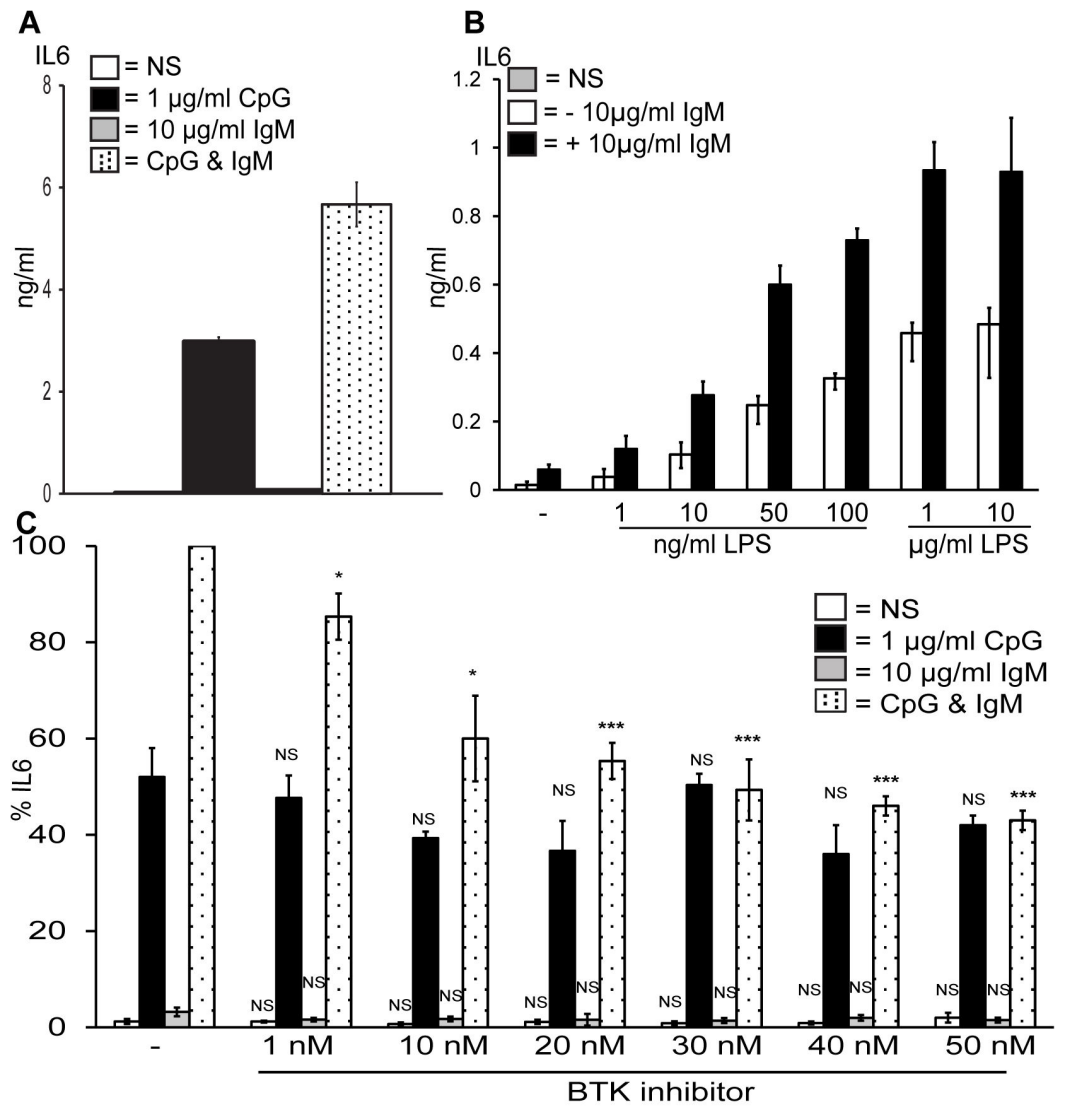
BTK is required for synergistic IL6 production in response to TLR9 and BCR stimulation in primary murine B cells. Primary murine B cells were isolated from the spleens of wild type mice, and stimulated with (A) CpG, anti-IgM or both for 48 hr, (B) LPS, anti-IgM or both for 48 hr at the concentrations shown or (C) pre-treated for 1 hr with the indicated concentrations of the BTK inhibitor and then stimulated with CpG, anti-IgM or both for 48 hr. The supernatants were then collected and IL6 production was measured by ELISA. (A) Data show mean ± standard deviation of a single experiment carried out in triplicate and are representative of three independent experiments. (B) Data show mean ± S.E.M of three independent experiments, each carried out in triplicate. (C) Data show mean ± S.E.M of three independent experiments each carried out in triplicate in which the concentration of IL6 produced by CpG and anti-IgM was set to 100% and all other simulations were normalised to this. IL6 levels induced maximally by CpG and anti-IgM ranged from 5–8.5 ng/ml across different mice. ***, p < 0.005, **, p < 0.01, * p < 0.05, not significant (NS) p > 0.05; significant differences between cells treated with DMSO control or BTK inhibitor and stimulated with CpG, anti-IgM or CpG plus anti-IgM.

We first confirmed the synergy previously seen in murine B cells in response to TLR9 and BCR stimulation [10]. We treated murine splenic B cells with CpG-DNA to stimulate TLR9, F(ab’)_2_ anti-mouse IgM (anti-IgM) to crosslink the BCR or both CpG and anti-IgM for 48 hr and examined IL6 production by ELISA. As shown in Figure 1A murine splenic B cells stimulated with 1 µg/ml CpG produced IL6 (black bar) and stimulation with 10 µg/ml anti-IgM resulted in very little IL6 production (grey bar). However, co-stimulation of the B cells with CpG and anti-IgM led to a doubling in the production of IL6 (black dotted bar) clearly indicating synergistic IL6 production in response to TLR9 and BCR activation. To examine if this was specific to TLR9 we next examined if the murine splenic B cells also synergised for the TLR4 ligand LPS and the BCR. We stimulated the B cells with increasing concentrations of LPS and this resulted in the production of IL6, although at a much lower level to that seen with CpG-DNA, as shown in Figure 1B (white bar). In response to anti-IgM again very little IL6 was produced by the B cells (black bar) and co-stimulation of the B cells with LPS and anti-IgM did result in enhanced IL6 production (black bars), although not to the same extent as in response to TLR9 activation. This data reveals that both TLR9 and TLR4 synergise with the BCR although the signal generated is much stronger in response to TLR9 activation suggesting that TLR9 and the BCR are especially competent at synergising.

We next investigated if BTK was required for the TLR9 and BCR synergy as the inhibitor is of interest in SLE therapy and TLR9 is linked to the progression of this autoimmune disease. Figure 1C illustrates that the BTK inhibitor concentration-dependently inhibited the production of IL6 in response to co-stimulation with CpG and anti-IgM (black dotted bars). The BTK inhibitor did not significantly block IL6 production in response to CpG alone (black bars) or anti-IgM alone (grey bars) indicating that BTK is required solely for the synergistic IL6 production in murine B cells in response to TLR9 and BCR activation.

Stimulation of TLR9 and the BCR results in synergistic IL6 production, up regulation of CD69, CD86 and MHC-class-II surface expression and BTK is required for this in primary human B cells

**Figure 2:**
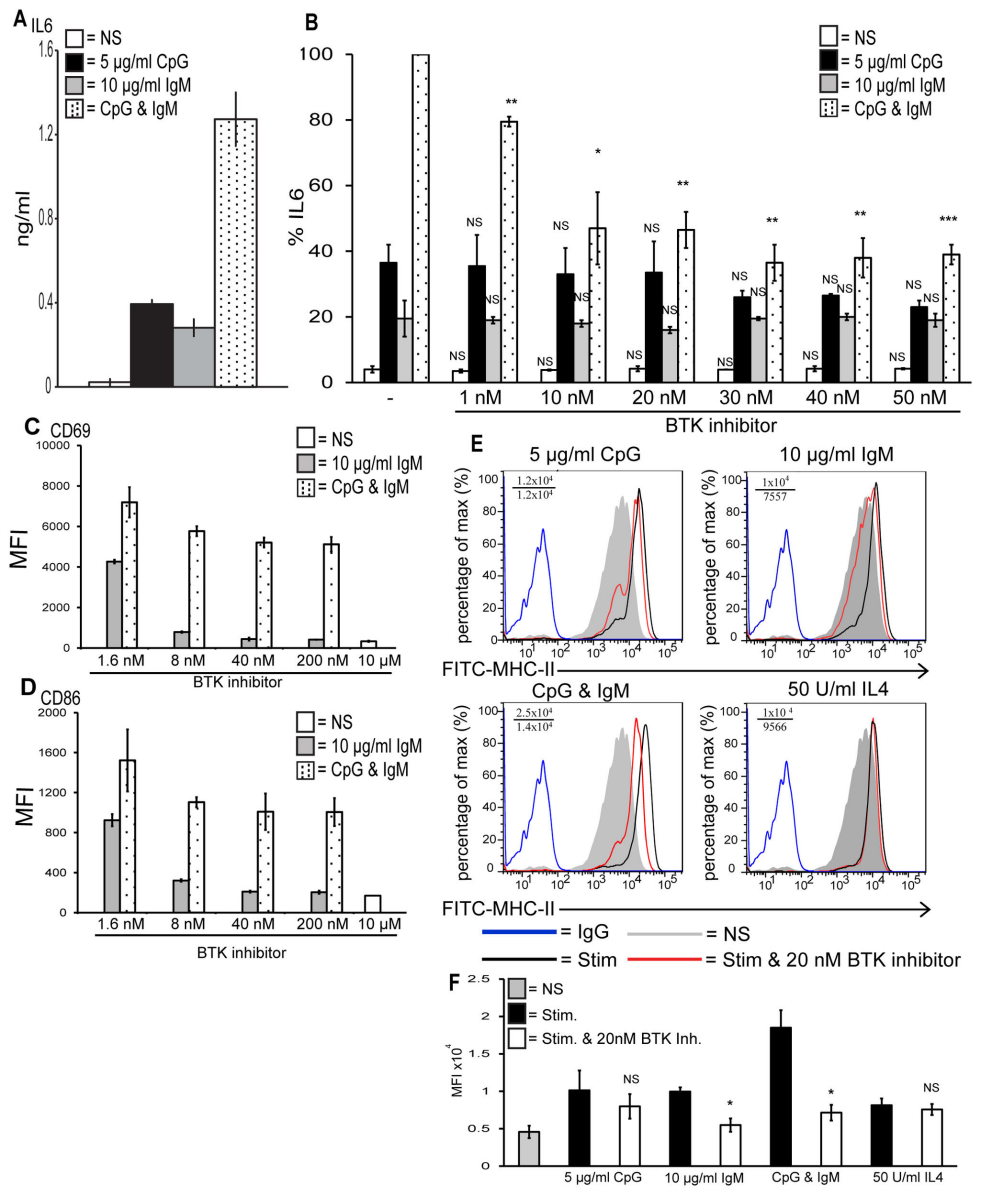
BTK is required for synergistic IL6 production and B cell activation in response to TLR9 and BCR stimulation in primary human B cells. Primary human B cells were stimulated with (A) CpG, anti-IgM or both for 48 hr, (B) pre-treated for 1 hr with the indicated concentrations of the BTK inhibitor and then stimulated with CpG, anti-IgM or both for 48 hr. The supernatants were then collected and IL6 production was measured by ELISA. (A) Data show mean ± standard deviation of a single experiment carried out in triplicate and are representative of three independent experiments. (B) Data show mean ± S.E.M of three independent experiments each carried out in triplicate in which the concentration of IL6 produced by CpG and anti-IgM was set to 100% and all other simulations were normalised to this. IL6 levels induced maximally by CpG and anti-IgM ranged from 1–2.5 ng/ml across different donors. ***, p < 0.005, **, p < 0.01, * p < 0.05, not significant (NS) p > 0.05; significant differences between cells treated with DMSO control or BTK inhibitor and stimulated with CpG, anti-IgM or CpG plus anti-IgM. (C–D) Primary human B cells were stimulated for 24 hr with anti-IgM or CpG plus anti-IgM, stained with an anti-CD69 antibody or with an anti-CD86 antibody and analysed by FACS. Median fluorescent intensities (MFI) are shown. Data show mean ± spread of biological duplicate determinations. Results are representative of two independent experiments. (E) Primary human B cells were stimulated for 48 hr with CpG, anti-IgM, both or IL4, stained with an anti-FITC-MHC-class-II antibody or with an anti-FITC-IgG isotype control antibody and analysed by FACS. Mean fluorescent intensities (MFI) of stimulated versus stimulated plus inhibitor are shown. Results are representative of three independent experiments. (F) The mean fluorescence intensities from three independent experiments were combined to confirm that the BTK inhibitor significantly affected surface expression of MHC-II. Data show ± S.E.M of three independent experiments.

We next examined TLR9 and BCR signalling in primary human B cells. The BTK inhibitor is in clinical studies for the treatment of several lymphonas and as such the role of BTK in human B cell synergy with TLR9 is of great therapeutic interest. As shown in Figure 2A primary human B cells produced IL6 in response to 5 µg/ml CpG (black bar) and in response to 10 µg/ml F(ab’)_2_ anti-human IgM (grey bar). The B cells stimulated with both CpG and anti-IgM produced a far great amount of IL6 (black dotted bar) again revealing synergy in B cells in response to TLR9 and BCR activation.

As illustrated in Figure 2B human B cells produced IL6 in response to CpG (black bars) and anti-IgM (grey bars) alone and the BTK inhibitor had no effect on this as the amount of IL6 produced did not change significantly. Conversely, the BTK inhibitor did significantly block the enhancement in IL6 production in the human B cells in response to both co-stimulation with CpG and anti-IgM (black dotted bars).

We then examined the ability of the BTK inhibitor to block B cell activation in response to anti-IgM alone or in conjunction with CpG stimulation. As shown in Figure 2C the BTK inhibitor blocked the up-regulation of CD69 on the surface of primary human B cells in response to anti-IgM alone in a concentration-dependent manner (grey bars). Stimulation of the cells with CpG and anti-IgM resulted in an increase in the level of CD69 on the surface and this was also reduced by the BTK inhibitor (black dotted bars). A similar result was seen when CD86 surface expression was examined as shown in Figure 2D. The BTK inhibitor blocked the up-regulation of CD86 in response to anti-IgM alone (grey bars) and CpG plus anti-IgM (black dotted bars). The BTK inhibitor did not reduce the level of surface expression of CD69 or CD86 in response to CpG alone (data not shown).

B cell receptor signalling is also known to be involved in the presentation of antigen to T cells through the translocation of the MHC-class-II molecule to the surface of the B cell in response to BCR activation. As such we next determined if BTK was required for this in primary human B cells. As shown in Figure 2E CpG stimulation resulted in an increase in the amount of MHC-II on the surface of the B cells (black line, top left panel) and pre-treatment of the B cells with 20 nM BTK inhibitor had little effect on this as the MFI only decreased by 14% (red line, top left panel). The B cells also up-regulated the level of MHC-II on their surface in response to anti-IgM stimulation (black line, top right panel) and the BTK inhibitor did reduce this by 31% (red line, top right panel) indicating that BTK is somewhat required for IgM-dependent MHC-II surface expression. Stimulation of the B cells with both CpG and anti-IgM resulted in a higher amount of surface expression of MHC-II than with either ligand alone (black line, bottom left panel) and this was decreased by 46% in the cells pre-treated with the BTK inhibitor (red line, bottom left panel). This revealed a requirement for BTK in TLR9 and BCR-dependent MHC-II up-regulation. IL4 was used as a control as it is known to induce the translocation of MHC-II to the surface of B cells [25]. This was seen to be the case (black line, bottom right panel) and the BTK inhibitor had no effect on this (red line, bottom right panel).

To confirm these data were significant the mean fluorescence intensities (MFI) from three independent experiments were analysed. As shown in Figure 2F the BTK inhibitor significantly reduced the levels of MHC-II surface expression in response to anti-IgM alone and CpG plus anti-IgM but not in response to CpG alone or the control ligand IL4.

### BTK is required for co-localisation of TLR9 and the BCR within human B cells

**Figure 3:**
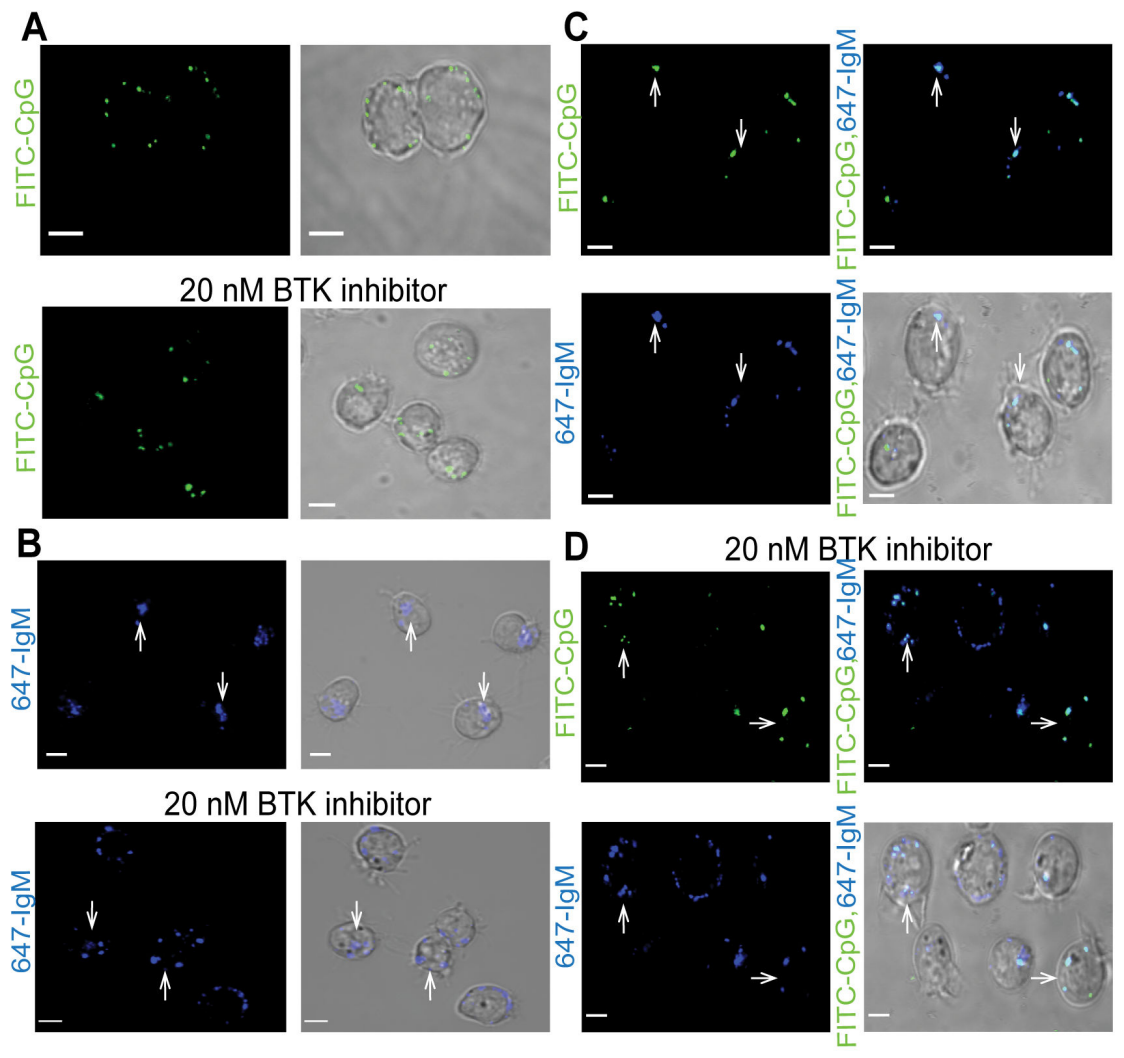
BTK is required for IgM-dependent co-localisation of TLR9 and the BCR in Namalwa B cells. Namalwa B cells were pre-treated with the BTK inhibitor for 1 hr and stimulated with (A) FITC-labelled CpG, (B) Alexa-fluor 647-labelled anti-IgM or (C) both for 16 hr. The cells were then examined by confocal microscopy. To confirm that BTK was required for this co-localisation 50 cells stimulated with CpG and anti-IgM were examined and the number of specks containing both proteins was counted yielding an average of 1.4 specks/cell. This was repeated for 50 cells pre-treated with the BTK inhibitor prior to CpG and anti-IgM stimulation and these cells contained an average of 3.5 specks/cell. A 2x2 contingency table chi-squared test was carried out on the total number of specks giving a *p* value of <0.001 indicating that TLR9 and the BCR were located in significantly more regions in the BTK inhibitor treated cells. Data are representative of at least four independent experiments (bars 5 µM) in which a minimum of 50 cells were examined.

We next examined the mechanism behind the role played by BTK in TLR9 and BCR synergy. The study by Chaturvedi et al. stated that in splenic murine B cells synergy occurs due to the co-localisation of TLR9 and the BCR within an auto-phagosome-like compartment that also contained p38, LAMP-1 and the invariant chain of MHC-class-II [10]. Thus we determined if BTK was required for this process using the human Namalwa B cell line as primary human B cells were unsuitable for imaging. As shown in Figure 3A CpG was localised to several regions of the Namalwa B cells (green areas, top panels) and pre-treatment of the cells with 20 nM BTK inhibitor did not change this pattern of localisation (green areas, bottom panels). As shown in Figure 3B the treatment of Namalwa cells with anti-IgM induced BCR translocation from the plasma membrane into one pool within the B cells (arrows, top panels). BTK was required for this pooling event as in the presence of 20 nM BTK inhibitor the pattern of BCR was drastically altered with several areas within the cells staining positive for anti-IgM (arrows, bottom panels).

The Namalwa cells were also stimulated with both CpG and anti-IgM and as shown in Figure 3C this led pooling of CpG to the area of the cell that also contained the anti-IgM (arrows, top right panel) indicating that TLR9 and the BCR co-localised within human B cells. Figure 3D revealed that pre-treatment of the cells with 20 nM BTK inhibitor changed the localisation of CpG and anti-IgM. While the two did co-localise this occurred in several distinct area of the cells and there was no obvious pooling of the CpG and anti-IgM to one area of the B cell (arrows, top right panel). Thus these data indicate that BTK is essential for co-localisation of TLR9 and the BCR within human B cells and this co-localisation may be necessary for synergistic signalling as suggested by Chaturvedi et al.

### PLC-γ2 and IP-3 are also required for TLR9 and BCR synergistic IL6 production in primary human B cells

**Figure 4:**
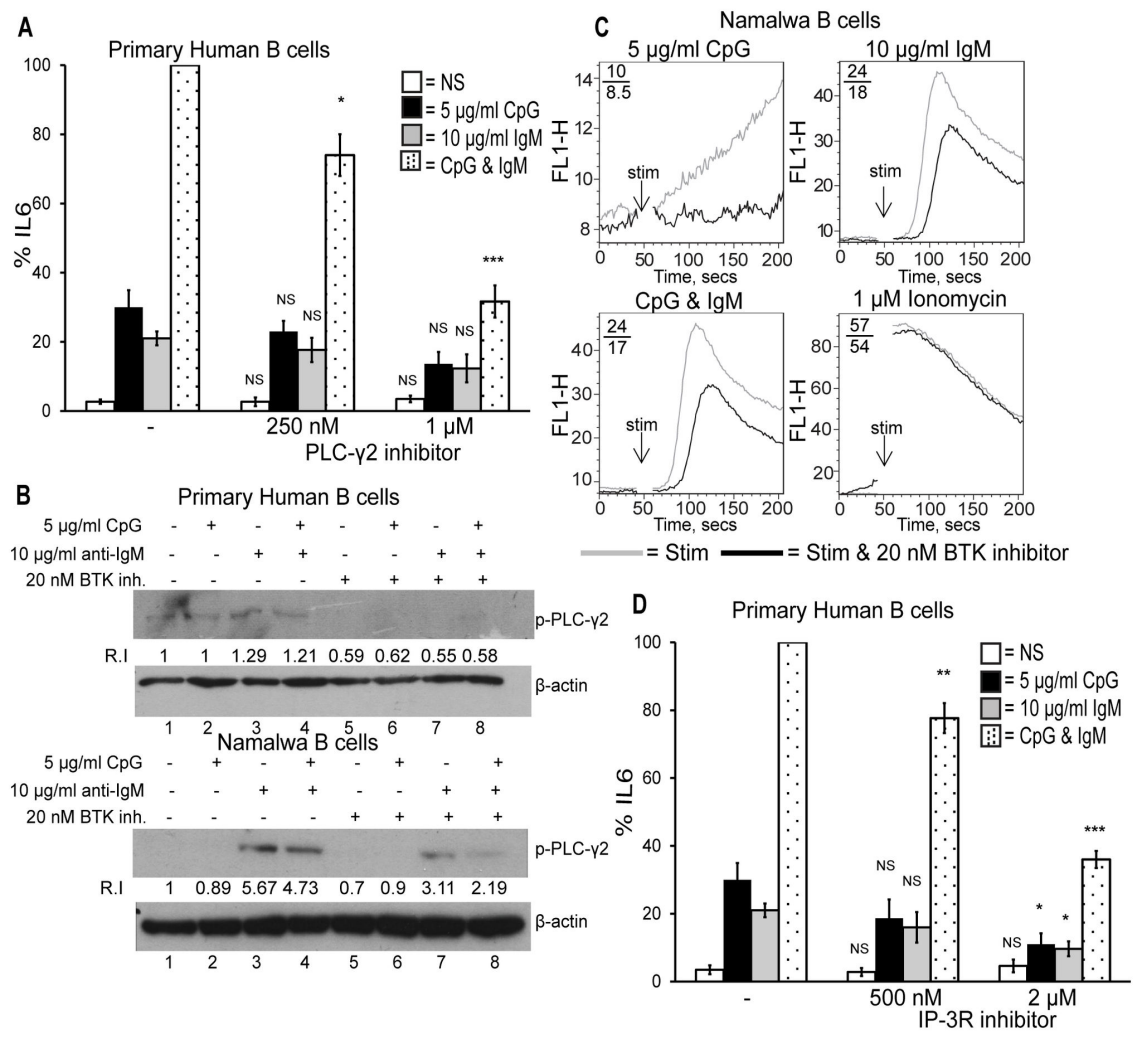
PLC-γ2 and IP-3 are required for TLR9 and BCR synergistic IL6 production. Primary human B cells were pre-treated for 1 hr with (A) the PLC-γ2 inhibitor U-73122 or (D) the IP-3 receptor inhibitor, Xestospongin C. The cells were then stimulated with CpG, anti-IgM or both for 48 hr. The supernatants were collected and IL6 production was measured by ELISA. Data show mean ± S.E.M of three independent experiments each carried out in triplicate in which the concentration of IL6 produced by CpG and anti-IgM was set to 100% and all other simulations were normalised to this. Results are representative of three independent experiments. IL6 levels induced maximally by CpG and anti-IgM ranged from 0.6–1.5 ng/ml across different donors. ***, p < 0.005, **, p < 0.01, * p < 0.05, not significant (NS) p > 0.05; significant differences between cells treated with DMSO control or BTK inhibitor and stimulated with CpG, anti-IgM or CpG plus anti-IgM. (B) Primary human and Namalwa B cells were incubated with the BTK inhibitor for 1 hr, stimulated with CpG, anti-IgM or both for 30 min and the cell lysates were examined for phospho-PLC-γ2 and β-actin by SDS-PAGE and Western Blot analysis. Densitometric analysis of band intensities was determined, where each band was normalised to its β-actin and the relative intensity (R.I.) of the bands over the non-stimulated control (in lane 1) were calculated. (C) Namalwa human B cells were incubated with the BTK inhibitor for 30 min. The cells were then incubated with Fluo-3AM, a fluorescent calcium indicator, for 20 min. Basal levels of calcium were measured for 40 sec, the cells were stimulated with CpG, anti-IgM, both or ionomycin and analysed by FACS for a total of 200 sec. Mean fluorescent intensities (MFI) of stimulated versus stimulated plus inhibitor are shown. Results are representative of three independent experiments.

We next sought to determine what was responsible for TLR9 and BCR synergy downstream of BTK with the aim of identifying the target of BTK that allowed the two signalling molecules to co-localise and enhance signal transduction. To this end we looked directly downstream of BTK on the BCR signalling pathway and examined the role of PLC-γ2 in synergistic IL6 production As shown in Figure 4A, similar to BTK inhibition, PLC-γ2 inhibition prevented the enhancement in IL6 production seen in response to CpG and anti-IgM (black dotted bars) whilst having no effect on CpG (black bars) or anti-IgM (grey bars) induced IL6 production. This was expected as PLC-γ2 is a direct target of BTK and as such is one step downstream of BTK on the BCR pathway involved in synergy.

We next examined the phosphorylation of PLC-γ2 in response to BCR activation. As shown in Figure 4B primary human B cells stimulated with anti-IgM and both CpG plus anti-IgM induced a low level of phosphorylation of PLC-γ2 (lanes 3-4, top panel) and the BTK inhibitor blocked this (lanes 7-8, top panel). CpG stimulation did not induce this phosphorylation (lane 2, top panel). Similarly in Namalwa B cells PLC-γ2 phosphorylation occurred in response to anti-IgM and CpG plus anti-IgM stimulation (lanes 3-4, third panel) and the BTK inhibitor blocked this (lanes 7-8, third panel). This confirmed that the BTK inhibitor blocked BTK enzymatic activity, PLC-γ2 being a direct target of BTK.

Phosphorylation of PLC-γ2 by BTK results in its activation and allows it to cleave its target phosphatidylinositol 4,5-bisphosphate (PIP-2) into diacylglycerol (DAG) and inositol trisphosphate (IP-3). IP-3 is involved in Ca^2+^ flux from the ER due to its interaction with the inositol trisphosphate receptor (IP-3R). We therefore next examined calcium as signal downstream of TLR9 and BCR activation.

As shown in Figure 4C stimulation of the Namalwa cells with CpG alone did not result in Ca^2+^ flux (grey line, top left panel). Stimulation of the cells with anti-IgM did result in Ca^2+^ flux (grey line, top right panel) that was inhibited by 25% in the BTK inhibitor treated cells (black line, top right panel) indicating that BTK was required for Ca^2+^ flux in BCR signalling. There was no synergy in Ca^2+^ flux in the Namalwa cells in response to CpG and anti-IgM (grey line, bottom left panel). The cells were also stimulated with the Ca^2+^ ionophore ionomycin as a control and this treatment did result in Ca^2+^ flux (grey line, bottom right panel) that the BTK inhibitor did not block (black line, bottom right panel).

We next examined if IP-3 was required for synergy and therefore regulating the Ca^2+^ flux seen in response to BCR activation. As illustrated in Figure 4D inhibition of the IP-3 receptor with Xestospongin C also significantly blocked the enhancement in IL6 production in response to CpG and anti-IgM (black dotted bars) at both concentrations tested. A slight effect on CpG (black bars), or anti-IgM (grey bars) induced IL6 production was seen which was only significant at the higher concentration of Xestospongin C suggesting a potential requirement for calcium in TLR9 and BCR signalling independent from their synergy.

### Calcium is essential for TLR9 and BCR synergy

**Figure 5:**
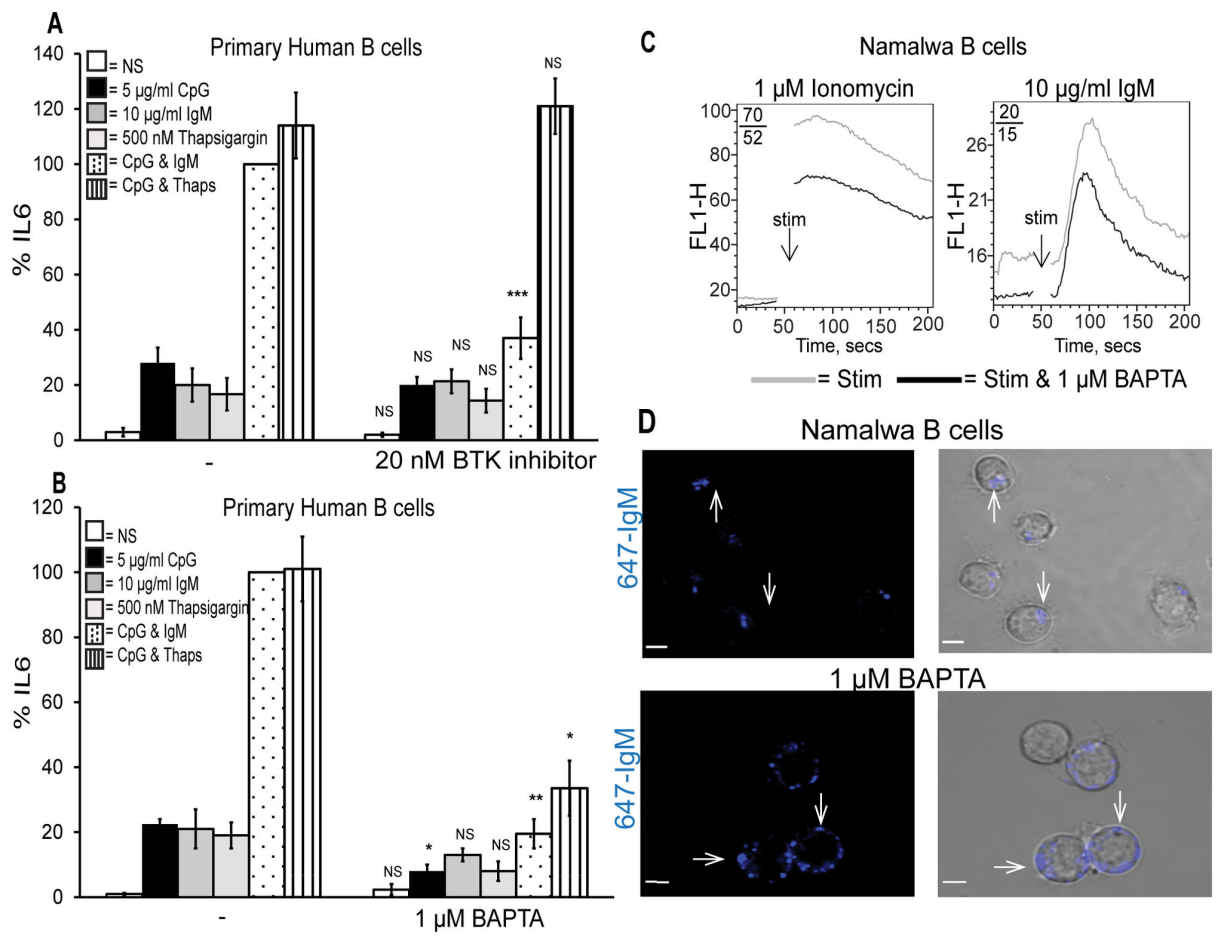
Calcium is required for TLR9 and BCR synergy. Primary human B cells were pre-treated for 1 hr with (A) the BTK inhibitor or (B) the Ca^2+^ chelator BAPTA and stimulated with CpG, anti-IgM, thapsigargin, CpG & anti-IgM and CpG & thapsigargin for 48 hr. Supernatants were collected and IL6 production was measured by ELISA. Data show mean ± S.E.M of three independent experiments each carried out in triplicate in which the concentration of IL6 produced by CpG and anti-IgM was set to 100% and all other simulations were normalised to this. IL6 levels induced maximally by CpG and anti-IgM ranged from 0.6-1.8 ng/ml across different donors. ***, p < 0.005, **, p < 0.01, * p < 0.05, not significant (NS) p > 0.05; significant differences between cells treated with DMSO control or BTK inhibitor and stimulated with CpG, anti-IgM, thapsigargin, CpG plus anti-IgM or CpG plus thapsigargin. (C) Namalwa B cells were pre-treated with BAPTA, incubated with Fluo-3AM and calcium flux was measured in response to the ligands indicated by FACS. Mean fluorescent intensities (MFI) of stimulated versus stimulated plus inhibitor are shown. (D) Namalwa B cells were pre-treated with BAPTA and stimulated for 16 hr with Alexa fluor-647-labelled anti-IgM. The cells were then examined by confocal microscopy (bars 5 µM) and a minimum of 50 cells were examined. To confirm that calcium was required for this pooling of the BCR 50 cells stimulated with anti-IgM were examined and the number of specks was counted yielding an average of 1.8 specks/cell. This was repeated for 50 cells pre-treated with BAPTA prior to stimulation and these cells contained an average of 5.5 specks/cell. A 2x2 contingency table chi-squared test was carried out on the total number of specks giving a *p* value of <0.001 indicating that the BCR was located in significantly more regions of the BAPTA treated cells. Results are representative of three independent experiments.

We further examined the requirement of calcium (Ca^2+^) for TLR9 and BCR synergy. Thapsigargin is a plant extract commonly used to raise cytosolic calcium levels as it prevents the removal of Ca^2+^ from the cytosol into the ER resulting in store depletion and influx of Ca^2+^ from the extracellular matrix. Figure 5A illustrates that stimulation of primary human B cells with 5 µg/ml CpG (black bar), 10 µg/ml anti-IgM (grey bar) or 500 nM thapsigargin (light grey bar) alone induced IL6 production that was not blocked by the BTK inhibitor. However, IL6 production was enhanced in B cells stimulated with CpG and thapsigargin and the BTK inhibitor was unable to block this enhancement (black dashed bars). These data indicate that a cytosolic Ca^2+^ increase allows for synergistic signalling. The BTK inhibitor was unable to prevent this influx of Ca^2+^ due to thapsigargin and hence did not block the enhanced IL6 production.

To further confirm the requirement of Ca^2+^ in TLR9 and BCR synergy we pre-treated the primary human B cells with the Ca^2+^ chelator BAPTA to remove any cytosolic Ca^2+^. Figure 5B reveals that Ca^2+^ chelation mirrored BTK inhibition. The enhanced IL6 production in response to CpG and anti-IgM was significantly blocked by BAPTA (black dotted bars). The BAPTA had a slight effect on the amount of IL6 produced by CpG (black bars), but no effect on the levels of IL6 produced in response to anti-IgM (grey bars) or thapsigargin (light grey bars) alone. The enhanced IL6 production in response to co-stimulation of the B cells with CpG and thapsigargin was also blocked by BAPTA (dashed bars) indicating again that an increased Ca^2+^ concentration in the cytosol was essential for TLR9 and BCR synergy.

To ensure that BAPTA was removing Ca^2+^ from the cytosol in the B cells we next analysed the Ca^2+^ flux of Namalwa B cells pre-treated with BAPTA. As demonstrated in Figure 5C BAPTA inhibited Ca^2+^ flux in response to ionomycin by 26% (left panel) and to anti-IgM by 25% (right panel). These results confirmed that Ca^2+^ was removed from the cytosol by the Ca^2+^ chelator BAPTA.

We also sought to confirm that calcium was required for pooling of the BCR within one compartment of the B cells. As shown in Figure 5D the Alexa-fluor-647-labelled anti-IgM localised to one compartment of the cells (arrows, top panels). This pattern was altered in the cells treated with BAPTA (arrows, bottom panels) with several distinct compartments containing the BCR. The data shown here demonstrates that downstream of BTK Ca^2+^ flux into the cytosol of B cells is necessary for synergistic IL6 production and localisation of the BCR to a single compartment with the B cells.

### The calcium sensor calmodulin is required for TLR9 and BCR synergy

**Figure 6:**
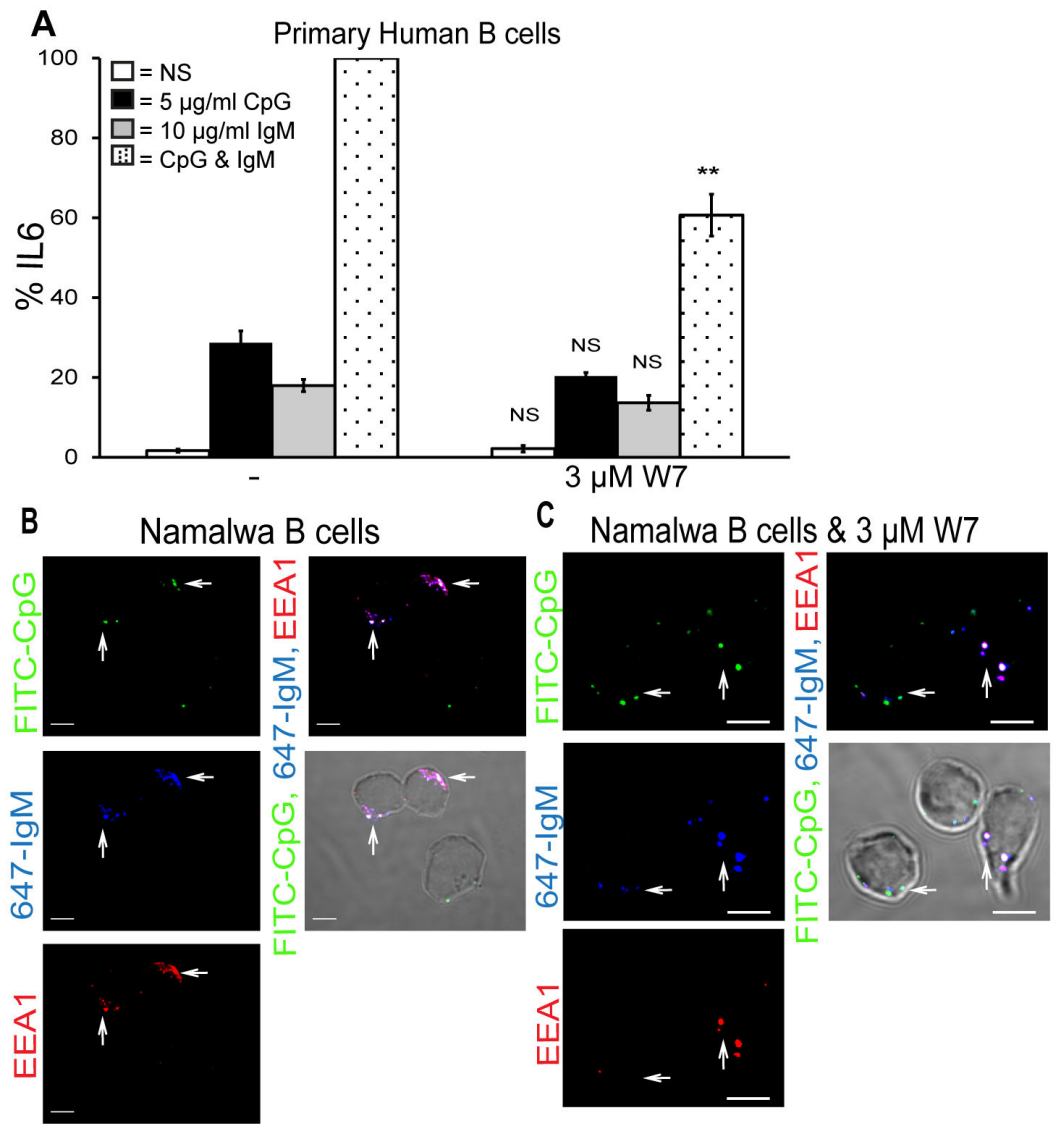
The calcium sensor calmodulin is essential for TLR9 and BCR synergy. (A) Primary human B cells were pre-treated for 1 hr with W7 and stimulated with CpG, anti-IgM, or CpG & anti-IgM for 48 hr. Supernatants were collected and IL6 production was measured by ELISA. Data show mean ± S.E.M of three independent experiments each carried out in triplicate in which the concentration of IL6 produced by CpG and anti-IgM was set to 100% and all other simulations were normalised to this. IL6 levels induced maximally by CpG and anti-IgM ranged from 2.5–4 ng/ml across different donors. ***, p < 0.005, **, p < 0.01, * p < 0.05, not significant (NS) p > 0.05; significant differences between cells treated with DMSO control or BTK inhibitor and stimulated with CpG alone, anti-IgM alone or CpG plus anti-IgM. Namalwa B cells were stimulated for 16 hr with FITC-CpG, Alexa fluor-647-labelled anti-IgM, in the absence (B) or presence of 3 µM W7 (C), fixed and stained for EEA1. The cells were then examined by confocal microscopy (bars 5 µM) and a minimum of 50 cells were examined. To confirm that calmodulin was required for the co-localisation of TLR9, EEA1 and the BCR 50 cells stimulated with CpG plus anti-IgM were examined and the number of fluorescent specks were counted yielding an average of 1.7 specks/cell. This was repeated for 50 cells pre-treated with W7 prior to stimulation and these cells contained an average of 4.6 specks/cell. A 2x2 contingency table chi-squared test was carried out on the total number of specks giving a *p* value of <0.001 indicating that TLR9, the BCR and EEA1 were located in significantly more regions of the W7 treated cells. Results are representative of three independent experiments.

Next we sought to identify a target of calcium. The proto-typical calcium sensor calmodulin was investigated as a potential target as it has been previously shown to be involved in the fusion of early endosome antigen 1 (EEA1) positive endosomes [26]. TLR9 is known to be localised to the early endosome with also contains EEA1 and as such we hypothesised that calmodulin could be required to traffic TLR9 to the BCR-containing compartment from which synergistic signalling occurs. To examine this we used a calcium-dependent calmodulin inhibitor W7 which binds the hydrophobic surface of calmodulin that is revealed upon Ca^2+^ binding. As shown in Figure 6A pre-treatment of primary human B cells with W7 significantly blocked the enhanced IL6 production in response to CpG and anti-IgM stimulation (black dotted bars). Calmodulin inhibition did not affect the amount of IL6 produced by CpG (black bars) or anti-IgM (grey bars) alone. This data suggests that calmodulin is the likely target of calcium involved in TLR9 and BCR synergy.

We finally sought to confirm that calmodulin was required for co-localisation of TLR9 and the BCR within one compartment of the B cells. As shown in Figure 6B in Namalwa B cells stimulated with both CpG and anti-IgM CpG was localised to one area of the cells (arrows, top left panel) as was IgM (arrows, middle left panel). EEA1 was also localised to the same region of the cells (arrows, bottom left panel). The overlay panel (top right) indicated that TLR9 and the BCR localise to an EEA1 positive compartment of B cells as shown by the white colour and the arrows. In the Namalwa B cells pre-treated with the calmodulin inhibitor W7 this localisation pattern was altered as shown in Figure 6C. The CpG was found in several distinct regions of the cells (arrows, top left panel) as was the IgM (arrows, middle left panel). EEA1 was also found in several regions of the B cells (arrows, bottom left panel). The overlay panel demonstrated that while the three molecules did still co-localise (arrows, top right panel) the pattern was altered with several distinct compartments containing TLR9, the BCR and EEA1.

Overall, therefore, our data reveal that the target of BTK in TLR9 and BCR synergy is ultimately the calcium sensor calmodulin. It is essential for enhanced IL6 production and co-localisation of TLR9 and the BCR within an EEA1 positive compartment of the B cells to allow synergistic signalling to occur.

## Discussion

Signal transduction pathways involving TLR9 and the BCR have been linked to the hyper B cell responses in autoimmune diseases such as Systemic lupus erythematosus (SLE) [27,28]. As such investigations into the mechanism behind this are of great importance with molecules that may specifically block this synergy of particular therapeutic interest. A novel small molecule BTK inhibitor, PCI-32765, is one such molecule of interest that has been shown to be highly specific as outlined below. It binds covalently to a cysteine residue (Cys-481) in the active site of BTK leading to irreversible inhibition of the enzymatic activity of BTK [23].

Several studies have been carried out using PCI-32765. When orally administered to mice with collagen-induced arthritis it reduced the level of circulating antibodies suppressing disease and in dogs with spontaneous B cell non-Hodgkin lymphoma (NHL) treatment with the BTK inhibitor induced clinical responses [23]. As abnormal BCR activation is linked to chronic lymphocytic leukaemia (CLL) the BTK inhibitor has also been examined as a tool to kill CLL cells. It has been shown to inhibit proliferation and promote apoptosis of CLL cells [29]. Orally administered, the BTK inhibitor induced significant objective responses in patients with B-cell NHL or CLL [30]. The BTK inhibitor has also been used as a tool to investigate the requirement of BTK in basophil activation revealing that BTK is required for IgE mediated responses [31]. Although this inhibitor is a highly potent and specific target for BTK, to date no investigations into the role of BTK in TLR and BCR synergy has utilised it as an experimental tool.

Using this novel small molecule inhibitor of BTK in our investigation into the mechanism of TLR9 and BCR synergy we have revealed that BTK is essential for synergistic IL6 production in response to CpG and anti-IgM in primary murine and human B cells. We used IL6 as a standard readout of B cell activation. IL6 production in response to TLR9 stimulation in B cells was independent of the enzymatic activity of BTK as TLR9-dependent IL6 production was not affected by the BTK inhibitor. This suggests that BTK can function downstream of TLR9 in B cells in a non-phosphorylated state. We also demonstrate that the BTK inhibitor blocks the up-regulation of the B cell markers CD69 and CD86 and the translocation of MHC-class-II to the surface of human B cells in response to CpG and anti-IgM. It also reduces Ca^2+^ flux into the cytosol of human B cells in response to anti-IgM. It is known that TLR9 and BCR synergy occurs due to the co-localisation of the two molecules within an auto-phagosome-like compartment upon BCR activation [10] and we demonstrate that BTK functions to allow this co-localisation to occur. Downstream of BTK we establish that Ca^2+^ flux into the cytosol of human B cells is also required for synergy.

Calcium is well recognised as a second messenger involved in endosome fusion [32] and much work has been carried out to identify the targets of calcium required for this fusion. One such target is calmodulin, a member of the E-F-hand family of Ca^2+^ -sensing proteins [33]. Calmodulin is proto-typical Ca^2+^ sensor and is involved in proliferation, growth and movement of cells [34,35]. As calmodulin has previously been shown to be involved in the fusion of EEA1 containing endosomes [26,36] and it is well documented that TLR9 is localised to EEA1 positive early endosomes [37,38] it is likely that the BTK-dependent calcium flux in B cells functions to activate calmodulin thus allowing synergy. Using the calmodulin inhibitor W7 we demonstrate a role for calmodulin in synergistic IL6 production and co-localisation of TLR9 and the BCR within an EEA1 positive compartment in human B cells. As several genes code for the calmodulin family members of interest calmodulin-deficient mice have not been generated and we were unable to further investigate this novel discovery in mice.

Classically the internalisation of the BCR was thought to lead to its degradation and was a method of extinguishing BCR induced signalling pathways [39]. However, it has subsequently been revealed that the BCR can signal once it has been internalised through a dynamin-dependent mechanism [40,41]. This supports our data in which we reveal that BTK is required for co-localisation of TLR9 and the BCR within an auto-phagosome-like compartment in the B cells resulting in synergy. In B cells pre-treated with the BTK inhibitor TLR9 and the BCR do still co-localise but in several distinct regions of the cells indicating that BTK is not involved in the initial dynamin-dependent internalisation step but only for the pooling of both receptors into the auto-phagosome-like compartment. Further investigations into the internalisation of the BCR and the recruitment of TLR9 would be helpful in fully elucidating the mechanism of synergy due to co-localisation of the receptors.

The BTK inhibitor used here provides a potential therapeutic agent to block the production of autoantibodies by self-reactive B cells in SLE which are thought to be activated by TLR9 and/or TLR7 sensing host nucleic acids [42–45]. We also found that the BTK inhibitor could block synergistic production of IL6 by TLR7 and the BCR (data not shown).

In vivo work has already been carried out using the BTK inhibitor in the MRL-Fas (lpr) lupus model where it inhibited autoantibody production and the development of kidney disease [23] and in a spontaneous murine model of lupus where it dampened humoral and cellular autoimmunity, as well as lupus nephritis [46]. Mice overexpressing BTK specifically in their B cells also develop SLE-like autoimmunity that can be alleviated by the BTK inhibitor [47]. These studies are supported by the data presented here where we have identified the mechanism by which BTK inhibition could have beneficial effects in SLE. As such it is the ideal target for further development in the treatment of SLE.

The essential requirement of Ca^2+^ for TLR9 and BCR synergy has been clearly demonstrated in this study. However Ca^2+^ is not a potential target in SLE treatment due to its role in an plethora of immune signalling pathways [18]. Another potential therapeutic target identified in this study is the Ca^2+^ -dependent protein calmodulin, the inhibition of which also blocked TLR9 and BCR synergy by interfering with the co-localisation of TLR9 and the BCR within an EEA1 positive region of the B cell. Calmodulin provides a better target for investigations as it is downstream of calcium and has a less central role in signal transduction.

Another area of interest with regard to the co-localisation of TLR9 and the BCR is the role of UNC93B. It is known to be involved in the trafficking of the endosomal TLRs including TLR9 [48]. Mutations in UNC93B result in deficient antigen presentation [49] and it is also up-regulated in B cells of SLE patients [50]. Examination of the interactions between UNC93B and BTK in B cells could potentially clarify the mechanism of translocation of TLR9 to the BCR further.

In conclusion, we describe the central role of BTK in Ca^2+^ -dependent TLR9 and BCR synergy and identify calmodulin as the potential downstream target of BTK required for TLR9 and BCR co-localisation. Taken together with the in vivo work using the PCI-32765 BTK inhibitor this work demonstrates that targeting BTK provides a valid therapeutic approach to treat SLE and other autoimmune diseases.
